# *“If you have light, your heart will be at peace”*: A qualitative study of household lighting and social integration in southwestern Uganda

**DOI:** 10.7189/jogh.13.04026

**Published:** 2023-04-14

**Authors:** Matthew Ponticiello, Edwin Nuwagira, Mellon Tayebwa, Joseph Mugerwa, Hellen Nahabwe, Catherine Nakasita, John Bosco Tumuhimbise, Nicholas L Lam, Matthew O Wiens, Jose Vallarino, Joseph G Allen, Daniel Muyanja, Alexander C Tsai, Radhika Sundararajan, Peggy S Lai

**Affiliations:** 1 Weill Cornell Center for Global Health, New York, New York, USA; 2 Mbarara University of Science and Technology, Mbarara, Uganda; 3 Department of Public Health, California State University East Bay, Hayward, California, USA; 4 Schatz Energy Research Center, California State Polytechnic University, Humboldt, Arcata, California, USA; 5 Centre for International Child Health, BC Children's Hospital, Vancouver, British Columbia, Canada; 6 Dept of Anesthesia, Pharmacology & Therapeutics, University of British Columbia, Vancouver, British Columbia, Canada; 7 Department of Environmental Health, Harvard T.H. Chan School of Public Health, Boston, Massachusetts, USA; 8 Harvard Medical School, Boston, Massachusetts, USA; 9 Massachusetts General Hospital, Boston, Massachusetts, USA; 10 Department of Emergency Medicine, Weill Cornell Medicine, New York, New York, USA

## Abstract

**Background:**

Expanding electrification and access to other clean and affordable energy, such as solar energy, is a critical component of the Sustainable Development Goals, particularly in sub-Saharan Africa where 70% of people are energy insecure. Intervention trials related to access or less polluting household energy alternatives have typically focused on air quality and biological outcomes rather than on how an intervention affects the end user’s lived experiences, a key determinant of uptake and adoption outside of a research setting. We explored perceptions of and experiences with a household solar lighting intervention in rural Uganda.

**Methods:**

In 2019, we completed a one-year parallel group, randomized wait-list controlled trial of indoor solar lighting systems (ClinicalTrials.gov NCT03351504) in rural Uganda where participants are largely relying on kerosene and other fuel-based lighting received household indoor solar lighting systems. In this qualitative sub-study, we conducted one-on-one, in-depth qualitative interviews with all 80 female participants enrolled in the trial. Interviews explored how solar lighting and illumination impacted participants' lives. We applied a theoretical model linking social integration and health to analyse dynamic interactions across aspects of study participants’ lived experiences. Sensors were used to measure daily lighting use before and after receipt of the intervention solar lighting system.

**Results:**

Introduction of the solar lighting system increased daily household lighting use by 6.02 (95% confidence intervals (CI) = 4.05-8.00) hours a day. The solar lighting intervention had far-reaching social implications with improved social integration and, consequently, social health. Participants felt that lighting improved their social status, mitigated the stigma of poverty, and increased the duration and frequency of social interactions. Household relationships improved with access to lighting because of reduced conflicts over light rationing. Participants also described a communal benefit of lighting due to improved feelings of safety. At the individual-level, many reported improved self-esteem, sense of well-being, and reduced stress.

**Conclusion:**

Improved access to lighting and illumination had far reaching implications for participants, including improved social integration. More empirical research, particularly in the light and household energy field, is needed that emphasizes the impacts of interventions on social health.

**Registration:**

ClinicalTrials.gov No. NCT03351504

Lighting has numerous individual physical and mental health benefits. Illumination from lighting has been shown to reduce injuries via traffic accidents [1] and adequate exposure to lighting is also a critical component of maintaining healthy physiological processes such as human circadian rhythm and sleep cycle [2,3]. Further, both artificial and natural light have proven effective at treating mood disorders and other mental health conditions such as seasonal affective disorder and depression [4-6]. Some studies have gone even further to demonstrate the impact of lighting and illumination at the communal level. These studies cite the potential of lighting and illumination to reduce crime [7,8] and improve perceived feelings of safety [9-11]. Despite the existing research on lighting interventions, they fail to consider a key component of human health – social health. Social health can be defined as our ability to interact and form meaningful relationships with others which is, in turn, reflected in greater social participation and connectedness [12]. Social health, although seldom discussed in the light and energy literature, has previously been reported to have significant impacts on health outcomes, including mortality [13,14].

Household energy intervention trials in low- and middle-income countries (LMICs) have focused on air pollutant exposure and conventional biological outcomes such as childhood pneumonia incidence, but rarely consider effects on end user’s lived experiences. These experiences, however, are likely an important factor in driving acceptability and uptake of an intervention and may also lead to other important outcomes. By exclusively studying biological or clinical outcomes, health research may fail to identify key psycho-social benefits of interventions that contribute to the holistic health and quality of life in communities. Empirical research is therefore needed across health research, and particularly so in household energy intervention trials, to elucidate the impacts of these interventions on social health.

While access to energy is abundant in high-income settings such as the United States because of widespread access to reliable electricity, energy poverty remains pervasive in LMICs [15]. More than 1.6 billion people globally lack reliable access to electricity, many of which are in sub-Saharan Africa where 70% of people are estimated to be energy insecure [16,17]. This, in turn, results in a dependence on stopgap sources of energy that are inefficient and often polluting. In areas without access to electricity, families rely on fuel-based lighting sources – kerosene lamps and lanterns, candles, open fires – for illumination. These sources of illumination can be a major sources of air pollution in homes and are not economically efficient solutions – being significantly more expensive per lumen of light compared to electric lighting sources, including solar. The operational costs associated with purchasing kerosene or candles for light compounded by potentially unreliable supply chains for them can lead to a rationing of light use in homes and suppressed demand for lighting services [18-21]. Assessments of the impact of household energy interventions have often worked to establish justification on grounds of health or economic benefit – usually in the context of cooking services – or on the “acceptability” of technologies via proxies for adoption. While social health is both a component of health and a likely factor in adoption and sustained use of intervention technologies, few studies have examined this as a potential outcome.

We conducted a qualitative sub-study to investigate the impact of lighting and illumination on psychosocial health and other aspects of participants’ lived experiences. This study was conducted as part of a randomized waitlist-controlled trial in rural Uganda where participants were randomized to receive indoor solar lighting systems provided by a local vendor. Quantitative results from the parent trial have been described previously [19,20]. Here, we present qualitative data from interviews with trial participants describing the impact of improved household lighting and illumination on psycho-social health.

## METHODS

### Study design and population

**Figure 1:**
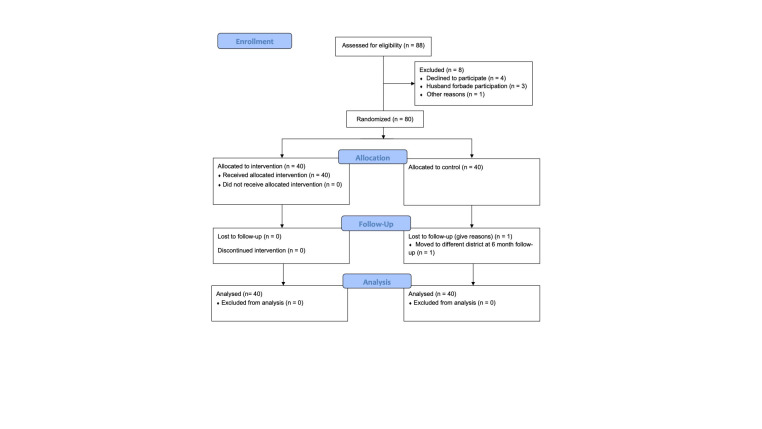
CONSORT flow diagram.

Between 2018 and 2019, we conducted a one-year parallel group, randomized waitlist-controlled trial of indoor solar lighting systems in Nyakabare parish, located in a rural region of southwest Uganda. Only 33% of Ugandans in this region have access to the electrical grid [22] though power outages are frequent. The study was powered to detect differences in household air pollution exposure as described previously [20]. Eighty adult women from distinct households were recruited from seven villages and randomized after enrolment targets were met in a 1:1 ratio stratified by primary lighting source using a random number generator (**Figure 1**). Women assigned to the intervention group received the intervention immediately, while women assigned to the waitlist-control group received the intervention after one year. At nine months following randomization – after the intervention group participants had received the intervention, but before the waitlist-control group had received the intervention – we began conducting qualitative interviews with all study participants.

### Ethics

Written informed consent was obtained from study participants. This study received ethical approval from the Mbarara University of Science and Technology (Protocol 02/11-16) and the Partners Human Research Committee (Protocol 2017P00306). Clearance to conduct the study was obtained from the Ugandan National Council of Science and Technology (Protocol PS 42) and the Ugandan President’s office. The study was registered with ClinicalTrials.gov under registration number NCT03351504.

### Procedures

The study intervention was an indoor solar lighting system that cost 565 000 Ugandan shilling (UGX) (approximately 150 US dollars (US$) at the time the study was conducted), obtained from a local vendor based in Mbarara township. These lighting systems were deployed in the intervention households between February and April 2018. The solar system consisted of a 30 W-peak (Wp) solar panel, 18 Amp-hour (Ah) battery, five Amp charge controller, four lighting points, installation services, and a two-year service warranty. Participants selected the location for each of the four bulbs in their homes. To evaluate the possibility that light rationing was present at baseline, we used sensors to monitor use of kerosene-based lighting and the intervention indoor solar lighting system as previously described [19].

### Data collection

One-on-one, in-depth qualitative interviews were conducted from January to July 2019 with all 80 participants enrolled in the clinical trial. A second subset of 21 interviews was also conducted to triangulate results via member checking. Overall, 101 interviews were completed and audio recorded.

Study interviews were conducted by four Ugandan research assistants (two male, two female) in the local language, Runyankole, and in a private setting at the participant’s home. An interview guide was created by a multidisciplinary group of Ugandan and US-based investigators in order to ensure consistent focus on core concepts, while allowing for exploration of novel concepts that emerged during the course of the study. Interviews explored the impacts of solar lighting on participants’ daily lives. Research assistants translated and transcribed each interview into English verbatim within 72 hours, and before conducting additional interviews. Translations were spot-checked by author MT, fluent in both Runyankole and English, in order to ensure the quality and fidelity of translations. PL reviewed transcripts for quality and content as they were generated.

### Data analysis

For qualitative data, following an inductive, thematic-analysis approach, all transcripts were reviewed to develop a coding scheme relevant to the trial outcomes, and to capture experiences of trial participants. Codes were independently developed by two authors (MP, RS) in vivo, through repeated engagement with the data set. Coding schema were compared, with discrepancies in codes resolved through discussion until a consensus was reached. Using a framework approach, coded data was organized by topic, and entered into an analytical matrix. Authors MP and RS reviewed the matrix to identify concepts which could organize the multi-level impacts of lighting and illumination on psycho-social health. We noted congruence between the social integration theoretical model (described below) and our conceptual findings, and therefore used this framework to guide organization of our study results.

For quantitative data, summary statistics are presented as median (interquartile range) for continuous variable, and categorical data are presented as number (%). Hours of daily lighting use was normally distributed. A linear mixed effects model as implemented by the nlme R package was used to compare sensor-based measurements of daily lighting use (in hours) before and after receipt of the intervention solar lighting system [23]. This model included only a binary term indicating whether the assessment was performed pre vs post intervention, with a random slope for subject to account for the longitudinal study design. Two-sided *P*-values less than 0.05 were considered significant. All quantitative data analysis was performed using R (version 4.1.1).

### Theoretical model

To examine intersecting factors that explain the role of light and illumination in shaping psychosocial health among participants in rural Uganda, we applied a theoretical model linking social integration and health to analyse dynamic interactions across aspects of lived experiences. Berkman, Glass, Brissette, and Seeman’s model of social integration and health was initially developed in the early 2000s summarizing the determinants of social network formation and the mechanisms through which social networks impact health [24]. Our application of the Berkman, Glass, Brissette, and Seeman model begins at the level of social-structural conditions (macro-level) and continues through social networks (mezzo-level), psychosocial mechanisms (micro-level) and pathways [25]. The socio-structural conditions (macro-level) include social change and major cultural, socioeconomic, and political factors that shape the nature of social networks. Social cohesion, conflict, and poverty are examples of socio-structural conditions. The next level is social networks, which are characterized by their sizes and structures, and include components of socialization such as frequency of face-to-face contact or duration of contact. Third, we examine psychosocial mechanisms of socialization (micro-level), which include social support, influence, and engagement. Psychosocial mechanisms then impact health through behavioural, psychological, and physiological pathways.

Our study provides evidence across levels of the Berkman, Glass, Brissette, and Seeman model to illustrate how household lighting influences social network formation, impacts social integration, and therefore health. By applying this model, we also underscore the growing importance of lighting and illumination as part of global health and development efforts.

## RESULTS

### Characteristics of qualitative study participants

A summary of characteristics of interview participants is shown in **Table 1**. The trial cohort was composed of 80 women aged 23-60 years old from seven different villages, the majority of whom were married or cohabiting, with no formal education or only a primary school level of education. Ownership of high-value household assets such as automobiles and motorcycles was rare. Most did not live in homes with cement walls or floors, nor did they have access to a ventilated/improved pit latrine. The majority of the day (~ 16 hours) was spent indoors, with four to five hours of self-reported light use daily.

**Table 1:** Characteristics of study participants*

	Overall	Control	Intervention	*P*-value
N	80	40	40	
Age, mean (SD)	39.7 (8.6)	38.1 (7.4)	41.3 (9.5)	0.091
Education (%)				0.754
*None*	11.0 (13.8)	4.0 (10.0)	7.0 (17.5)	
*Primary one-two*	22.0 (27.5)	12.0 (30.0)	10.0 (25.0)	
*Primary three-six*	28.0 (35.0)	15.0 (37.5)	13.0 (32.5)	
*Primary seven*	19.0 (23.8)	9.0 (22.5)	10.0 (25.0)	
Marital status (%)				0.602
*Married*	71.0 (88.8)	35.0 (87.5)	36.0 (90.0)	
*Cohabiting*	8.0 (10.0)	4.0 (10.0)	4.0 (10.0)	
*Separated/divorced*	1.0 (1.2)	1.0 (2.5)	0.0 (0.0)	
Owns a radio (%)	62.0 (77.5)	31.0 (77.5)	31.0 (77.5)	1.000
Owns a TV (%)	12.0 (15.0)	6.0 (15.0)	6.0 (15.0)	1.000
Owns a refrigerator (%)	1.0 (1.2)	0.0 (0.0)	1.0 (2.5)	1.000
Owns a motorcycle (%)	10.0 (12.5)	6.0 (15.0)	4.0 (10.0)	0.735
Owns a car (%)	1.0 (1.2)	0.0 (0.0)	1.0 (2.5)	1.000
Access to a ventilated improved pit latrine (%)	2.0 (2.5)	1.0 (2.5)	1.0 (2.5)	1.000
Home has cement walls (%)	12.0 (15.0)	3.0 (7.5)	9.0 (22.5)	0.117
Home has cement floors (%)	18.0 (22.5)	6.0 (15.0)	12.0 (30.0)	0.181
Wealth quintile, mean (SD)	3.0 (1.3)	2.9 (1.2)	3.1 (1.5)	0.562
Primary lighting source (%)				0.987
*Kerosene*	33.0 (41.2)	16.0 (40.0)	17.0 (42.5)	
*Candles*	2.0 (2.5)	1.0 (2.5)	1.0 (2.5)	
*Flashlight*	5.0 (6.2)	3.0 (7.5)	2.0 (5.0)	
*Solar*	27.0 (33.8)	14.0 (35.0)	13.0 (32.5)	
*Electrical bulbs (national grid)*	13.0 (16.2)	6.0 (15.0)	7.0 (17.5)	
Secondary lighting source (%)				
*Kerosene*	53.0 (66.2)	27.0 (67.5)	26.0 (65.0)	1.000
*Candles*	8.0 (10.0)	5.0 (12.5)	3.0 (7.5)	0.709
*Flashlight*	3.0 (3.8)	1.0 (2.5)	2.0 (5.0)	1.000
*Solar*	29.0 (36.2)	14.0 (35.0)	15.0 (37.5)	1.000
*Electrical bulbs (national grid)*	9.0 (11.2)	5.0 (12.5)	4.0 (10.0)	1.000
Cooking fuel type (%)				0.500
*Charcoal*	3.0 (3.8)	1.0 (2.5)	2.0 (5.0)	
*Firewood*	76.0 (95.0)	39.0 (97.5)	37.0 (92.5)	
*LPG / natural gas*	1.0 (1.2)	0.0 (0.0)	1.0 (2.5)	

SD – standard deviation, LPG – liquefied petroleum gas

*Only women were recruited for this study.

Sensor-based measurements of daily lighting use for primary kerosene lighting users showed that prior to the intervention, average daily lighting use was 2.3 (interquartile range (IR) = 1.8-2.9) hours, whereas after the intervention, average daily lighting use was 8.3 (IR = 5.1-11.5) hours a day. In mixed effects models, introduction of the solar lighting system increased daily household lighting use by 6.0 (95% confidence interval (CI) = 4.0-8.0) hours a day, supporting the hypothesis that significant light rationing and suppressed demand was present at baseline prior to the introduction of the intervention solar lighting system.

### Qualitative results

**Figure 2:**
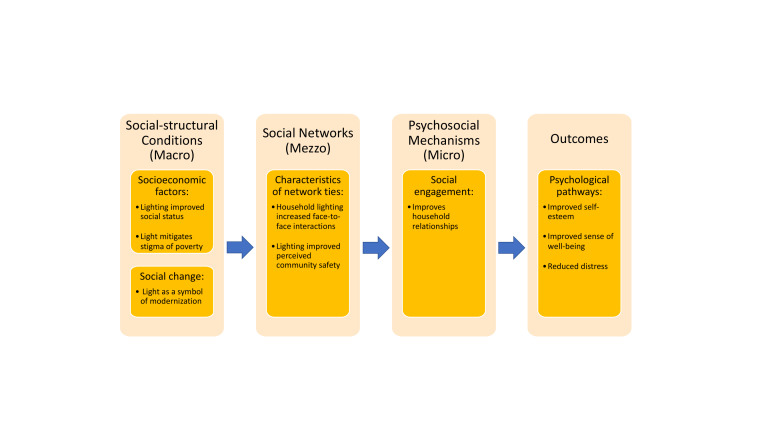
A modified Berkman, Glass, Brissette, and Seeman conceptual model elucidating the impact of light on social integration and health.

During the qualitative interviews, several themes emerged about the impacts of light and illumination on social integration and psycho-social health of participants, their families, and their communities in this study. We mapped these major themes to the socio-structural conditions (macro-level), social networks (mezzo-level), psychosocial mechanisms (micro-level), and Pathways domains proposed by Berkman, Glass, Brissette, and Seeman’s model linking social networks and health (**Figure 2**). The themes within this rubric consist of (1) socio-structural conditions: lighting improved social status, mitigated the stigma of poverty, and was considered a symbol of modernization; (2) social networks: household lighting and illumination increased interpersonal interactions and improved perceived community safety; (3) psychosocial mechanisms: lighting improved household relationships; and (4) outcomes: household lighting improved self-esteem, sense of well-being, and reduced stress. We present our results below organized by the domains of the Berkman, Glass, Brissette, and Seeman model, with quotations illustrating key concepts on the impacts of household lighting among our participants.

#### Socio-cultural conditions (macro)

Household lighting was described as having numerous impacts on socioeconomic factors and reflect socio-cultural conditions. Lighting was described as a factor in improving one’s social status and mitigated the stigma of poverty. In addition, household lighting was described as a source of pride because it indicated that one’s community was modernizing.

##### Socioeconomic factors

Having solar lighting was described as a quality worthy of respect, with social status being enhanced after gaining access to solar lighting. Participants noted that if their homes were illuminated, this would convey their elevated status to people from other communities.

*(When you have light) you even get respect from other people that come from far away ...you get respect. They know that you are not badly off, that you are well off! Intervention arm participant, Village 1, 28 years old*

Given its apparent visibility, especially compared to other forms of household energy, the household solar lighting system was considered a very public symbol. This, in turn, garnered further admiration from others and improved individual’s status by many in the community.

*When you put a panel on your roof, someone will see it from a distance and complement that house that has a panel. Even at night, if someone is very far and they look in this direction of our house. They will say “wow, that lady also got solar…”*

*Control arm participant, Village 2, 33 years old*

In addition, having access to renewable energy positioned participants in an “elevated” status, by helping others in their communities charge mobile phones.

*Yes, I am now on the level of other people, and even beyond others. Because in the past if we wanted to charge a phone, we would go to other people’s homes … Now that we have a solar that can help us to charge, we can even help others to charge.*

*Intervention arm participant, Village 1, 50 years old*

While lack of lighting and darkness were understood as markers of impoverishment, lighting was seen as a symbol of wealth. Some participants expected that lack of lighting draws associations with being poor and “lazy” from their community:

*We would be badly off (without lighting). People would see us as the poorest in the village. They would laugh at us like we are not worth living. People would regard us as the laziest since we are unable to do anything (without light).*

*Control arm participant, Village 3, 36 years old*

*If I didn’t have a lighting source, they would look at me as a needy or vulnerable person. Because if you don’t have a lighting source or a (kerosene) lamp or not even a small torch, it means you are a very poor person ... why would they come to your home? To get what from you? They take you as a worthless person.*

*Intervention arm participant, Village 4, 41 years old*

Living without household lighting also led to isolation as other community members were reticent to socialize with households without lighting.

*It is shameful because no one can come to your home in the evening when you are in total darkness. They actually fear (your home). And you expect someone to visit you when you are in total darkness? Not really. It is shameful.*

*Intervention arm participant, Village 3, 46 years old*

As such, having lighting was an important symbol to demonstrate success and wealth within villages:

*When you come at night to this village, (everyone) will see that we have light. We see that we are different from others. Some people are in darkness. But we are in the light.*

*Intervention arm participant, Village 4, 72 years old*

##### Social change

Light from lightbulbs was considered by many to be a sign of “modernity.” Kerosene lamps and candles were perceived as behind the times. Consequently, conceptually, using light bulbs began to represent a major social shift towards development. Participants noted that seeing another’s lights would make you want to modernize as well.

*These days modernity is taking over in terms of light. When they are conversing and he is seeing clearly, he gets interested in buying his own solar so that his home can look good.*

*Control arm participant, Village 5, 49 years old*

*I also wanted to be modernized. It’s a dot com era.*

*Control arm participant, Village 5, 31 years old*

#### Social networks (mezzo)

Light was seen to have significant impacts within social networks through increasing frequency, duration, and quality of socialization, thus improving social cohesion.

##### Characteristics of network ties

Participants reported lack of household impeded social gatherings after dark because people could not see one another.

*If you get a visitor, you can sit and have a conversation when there is light. You cannot sit and talk in darkness.*

*Intervention arm participant, Village 4, 41 years old*

With improved access to household lighting, people were able to socialize with their friends and neighbours with increased hours for socialization.

*I am sure that when I have light, I will joyfully and confidently welcome every visitor that comes.*

*Control arm participant, Village 4, 42 years old*

Participants described how neighbours would gather outside to socialize, using the solar lighting to illuminate the gathering. In this sense, access to lighting was something the whole community benefited from because it was shared.

*My friend, she is in the study as well, the neighbors expect her to put on the outdoor light in the evening. They use her place as a trading center to meet and other things, so her outdoor light helps them a lot.*

*Intervention arm participant, Village 6, 36 years old*

Lighting was also described as facilitating religious studies and gatherings, a central component of life for many participants. The ability to facilitate access to solar energy and lighting was described by one participant as “one of God’s miracles”:

*I need solar lighting because I want my kids to read and being able to congregate with my fellow born again Christians. With solar light we can pray that God does miracles for us. That’s my only desire. I also have friends who may need power to charge their phone. Because the good lord has done miracles for me, then I can also charge (their phones) for them.*

*Intervention arm participant, Village 6, 33 years old*

Household lighting was also described to have multiple positive effects on community safety. Participants explained that thieves would often come at night in the absence of lighting but were deterred by outdoor “safety” lights.

*Another thing that makes the outdoor light important is that even thieves fear to go to a place that has light. They will think that someone might see them from the inside or from the neighborhood.*

*Control arm participant, Village 4, 31 years old*

Both the owner of the solar lights and their neighbours experienced the benefits of lighting to improve community safety.

*I have the security light and you my neighbor has no solar or light at all. I switch on the security bulb and we all are able see.*

*Control arm participant, Village 5, 33 years old*

*If you switch on the security bulb the light can also light up at the neighbors place so that helps them in some way.*

*Control arm participant, Village 7, 39 years old*

#### Psychosocial mechanisms (micro)

Within the micro-level psychosocial mechanisms impacted by light, participants described how household light and illumination improved interpersonal relationships, including household relationships.

##### Social engagement

Non-renewable sources of household lighting in rural Uganda include candles and kerosene lamps. These lighting sources were described as a source of interpersonal conflicts if perceived as being used wastefully.

*In the past, whenever we would light paraffin (candle), my husband would shout and tell us to turn off the tadooba (kerosene lamp) claiming that we were finishing up the paraffin.*

*Control arm participant, Village 3, 34 years old*

Purchasing candles and kerosene for lamps was also costly, causing conflict over money to purchase fuel. Many participants explained that once they had access to solar lighting, they no longer needed to ask their partners for money for lighting expenditures, thus reducing conflicts.

*There will be no more shouting for money to buy paraffin. (My husband) is tired of us asking him for money every now and then.*

*Control arm participant, Village 1, 49 years old*

*Now that we have lighting, I will not make fun at him like “Mister, today you didn’t bring a candle home. How do you want us to survive?” We would make that as a joke. If we have light and we can see well where we are and the children can read their books, then why should I disturb my husband?*

*Control arm participant, Village 2, 33 years old*

Many described how family relationships in general improved with access to household lighting. Some women explained that arguments would arise amongst family members over who got to use the light source because there were not enough for the whole family. However, once given access to solar lighting, the conflicts ceased.

*Fighting for the one tadooba, taking it here and there. Then you hear the kids fighting, each one wants to have light. That story will be over.*

*Control arm participant, Village 1, 49 years old*

Finally, participants described a sense of relief with solar lighting, which reduced the financial burden of purchasing non-renewable lighting fuels. In addition, all members of the household benefited from improved household lighting to support completion of schoolwork and reading activities.

*The solar lighting made us all happy as a family. My children benefit a lot from it when reading their books. I personally am happy with the solar lighting and so is my husband. The money he used to spend on paraffin, he uses it to do other things.*

*Intervention arm participant, Village 7, 35 years old*

#### Outcomes

At the individual level, many participants described perceived psychological benefits of light. First, household lighting was described to improve individuals’ self-esteem. Household lighting also contributed towards a sense of improved well-being and reduced stress.

##### Improved self-esteem

Participants report prior feelings of jealousy when they would see other homes with lighting when they did not and suggest feeling not at the “standard” of others. However, once provided with household solar lighting as a part of this study, these feelings were lifted.

*When I would walk around at night and see houses with better lights, it would annoy me. I would feel jealous of what they have because I wished to have better lights like they do. When I was here, the study brought me solar lighting and I was very happy. I don’t need to be jealous anymore. The solar lighting put me on their standard.*

*Intervention arm participant, Village 2, 62 years old*

Others reported that having household lighting allowed them to be less dependent on their neighbours for lighting sources. This newfound self-reliance led to feelings of happiness and pride.

*We also feel happy and proud to have our own (light), other than inconveniencing other people.*

*Intervention arm participant, Village 4, 28 years old*

##### Improved sense of well-being

Participants explained that having household lighting was a positive influence on their sense of well-being.

*When you have a lighting source you cannot be disturbed by anything. You feel good, like other people.*

*Control arm participant, Village 7, 38 years old*

Additionally, participants described the explicit feelings of joy associated with having household lighting.

*I will be very happy (when I have light). I will be happy in the house; I will be happy outside my house. The children will have to read the books without being disrupted. I will be here loving myself.*

*Control arm participant, Village 2, 33 years old*

##### Reduced distress

Lack of lighting was described as a paralyzing force in participant’s lives, necessitating early bedtimes and forcing people to stay in their homes for fear of dangers in the darkness.

*I would be miserable really because of the darkness. I think I would even be sleeping very early because you can’t stay awake when it is dark. If you don’t have light you tend to fear. You can’t move. You can’t go outside.*

*Intervention arm participant, Village 4, 59 years old*

Participants were worried about snakes or other dangerous animals lurking in the dark, as well as hazards within their own homes. That worry was alleviated with the receipt of the solar lighting system, and they were able to achieve peace of mind.

*If you are in darkness and a snake comes to your house, it will bite you. Maybe the child defecates in the house, and it is dark everywhere... you will end up stepping in it. It is very bad. You don’t know what is in front of you. Your heart will race and you can’t settle down ... always worried about what may happen. But if you have light, your heart will at peace, and you’ll feel at home.*

*Intervention arm participant, Village 4, 41 years old*

Household lights also included outdoor security lights used to ward off invaders and thieves. Participants explained that this made them feel safer at night, contributing a sense of peace and safety at home.

*I was also safe with the security lights. Sometimes bad people tried to kill our dogs, so that they can steal our goats and sheep. When I put on light, I feel safer.*

*Control arm participant, Village 5, 35 years old*

##### Modified conceptual model

Our results can be used to modify the Berkman, Glass, Brissette, and Seeman model in such a way that links access to light with social integration and health (**Figure 2**). We found that lighting primarily influences two domains of social-structural conditions: (1) socioeconomic factors and (2) social change. Light as a marker of social status reflects a newfound symbol of wealth that ameliorates the stigma of poverty, while light as a representation of modernization denotes a social change towards development – both of which underpin the conditions that form social networks. Further, participants described significant changes to their social networks via shifted characteristics of network ties due to light. Light facilitated social interactions for many, and participants reported shared safety benefits of a neighbour owning lighting. These benefits of light reflect improved frequency of face-to-face contact, duration of socialization, and reciprocity of ties. Social engagement, a psychosocial mechanism of social integration, also improved at the household level due to lighting. Participants described reduced conflicts over lighting with access to solar light which, in turn, strengthened their relationships with their partners and families. Lastly, there were numerous psychological pathways, which we modified to outcomes, that improved with access to light, including sense of well-being and reduced stress.

## DISCUSSION

In this qualitative study embedded within a randomized trial of a lighting intervention in rural Uganda, we identified multiple ways in which improving the quantity and quality of household lighting influenced study participants’ social network formation and social interactions, and thus improved social integration and subsequently psycho-social health. Our data revealed that access to lighting improved social status for participants and ameliorated the stigma of poverty. Improved lighting services facilitated more interpersonal interactions between community members, strengthened perceived community safety, and improved household relationships. Lastly, participants reported that lighting improved their self-esteem, sense of well-being, and reduced psychological stress. There is little in the household energy and lighting literature focused on social health impacts thus highlighting a gap in how these programs are evaluated. Were these social outcomes to be accounted for in intervention trials, it is likely that the resulting cost-benefit ratios would be even more favourable than current estimates indicate [19,26,27].

Currently, there is a major gap in the household energy literature related to the impact of lighting and illumination on social integration and health. In the study site of rural southwestern Uganda, we found that improved lighting services promoted social integration across scales and, as a result, improved psycho-social health. At the macro-level, lighting improved participants’ social status; at the mezzo-level lighting improved social cohesion and engagement; and at the individual level lighting directly improved self-reported psychological well-being. While other studies note the profound health benefits of social integration [13,25,28-34], no household energy research we identified consider light as a facilitator of social integration to improve health. Typically, household energy intervention studies have focused on reducing household air pollution to improve biological and clinical outcomes [21,28,35,36] and studies in lighting have looked at the explicit connection between lighting and physiological processes [2,3]. This gap in the literature suggests a need to expand the scope of household energy evaluation to include a broader array of outcomes that capture participants’ lived experiences, especially considering the World Health Organization’s definition of health as encompassing “social well-being” [37].

Outside of health research and the clinical consequences of light and lighting sources, social scientists have begun to examine the political-economic and socio-cultural dimensions of access to and development of lighting technologies [38,39]. Some have studied lighting sources through a lens of meaning-making and development discourse to understand lighting, and specifically solar lighting lamps, as a humanitarian good and as an “agent of social development” [40]. The understanding of light as an agent of social development is also reflected in our data as many participants equated light to modernization. Other social scientists have explored the socio-technical barriers to implementing light-providing technologies in low-income settings, noting intra-communal jealousy, theft and vandalism as barriers to implementation [41]. This finding somewhat parallels our data that revealed access to lighting can be a status symbol because it shows that those without this status symbol may be jealous and covet it. Further, while our study did not include men, others have documented the gendered ownership and control over household lighting sources that often disempowers women [42,43]. While the aforementioned studies present an opportunity to contextualize our findings within the social science literature, it remains clear that social science and clinical perspectives on household lighting are disjointed, and this may be the very first study that begins to apply a transdisciplinary research approach to understand the social value of light through its impacts on social integration and health [44].

Our study’s findings are also resonant with the literature on the social meaning of energy poverty. A previous study in Afghanistan found that lack of access to electric light was interpreted by marginalized communities as a reminder of perceived exclusion and discrimination, as well as the feeling that they have been left behind by the international community [45]. Another study in Zimbabwe had similar findings, with many participants describing the social suffering associated with energy poverty and lack of illumination. In fact, one participant noted, “Who would want to be associated with darkness? Living in darkness shows that we are really backward” [46]. The sentiment that a lack of lighting was accompanied by social suffering and considered a sign of underdevelopment echoes our own finding that darkness is “shameful”, and that lighting is both a status symbol and a sign of modernization. Consequently, it is imperative that global health and development efforts begin to understand access to energy and light not just as a method to reduce air pollution or a Sustainable Development Goal, but rather a eudemonic pursuit that centres communities’ and individuals’ psycho-social well-being [47,48].

Our study has a few limitations. First, all trial participants were women, and our findings may not apply to men. In this particular study setting of rural Uganda, the household division of labour is such that women are responsible for food preparation, child rearing, and household management – so the burden of food insecurity, water insecurity, and energy insecurity (and their disparate impacts on health and well-being) is highly gendered [46,49]. Thus, our data on the social consequences of light and energy poverty are derived from the social group most adversely affected by it. Second, it is possible that intervention participants may have felt pressured to express positive opinions of lighting after being given free solar lighting. To ensure that this aspect of the study design did not bias our data, we conducted the qualitative interviews after the intervention group had received the intervention and before the waitlist-control group had received the intervention. We found no differences in the data according to intervention group assignment, providing some reassurance that the potential pressure to express positive opinions did not exert significant influence on the data. It is possible that control arm participants may have also felt pressured to exaggerate the value of light to ensure that our study team followed through with our contractual obligation to provide the intervention. However, our study team has conducted patient-oriented and intervention research in this area for more than a decade with no such breaches to date, so we believe it is unlikely that the waitlist control participants would have approached participation in the trial with such an orientation. Furthermore, our research assistants were provided with thorough training on how to collect qualitative data with open-ended questions and follow-up probing, and they were closely supervised by an experienced team of investigators including an anthropologist with experience conducting qualitative investigations in this study setting. We also specifically probed about potential negative aspects of solar lighting but did not obtain significant data in this regard. Finally, qualitative data are highly contextual and may not apply to other settings. Other groups assessing the value of lighting and energy interventions should consider including men and/or collecting quantitative data on social integration and health to confirm our findings and consider longitudinal impacts of having lighting over the course of years.

Our work begins to fill a gap in the scientific literature and attempts to bridge social science and clinical research perspectives in household energy intervention trials, particularly in resource-limited settings. Access to lighting has far-reaching psycho-social health implications and should be considered a priority in global health and development efforts. The potential for light to improve social integration may lead to downstream effects on other health outcomes like mortality and should be further studied.
